# Road Traffic Sign Detection Method Based on RTS R-CNN Instance Segmentation Network

**DOI:** 10.3390/s23146543

**Published:** 2023-07-20

**Authors:** Guirong Zhang, Yiming Peng, Hai Wang

**Affiliations:** School of Automotive and Traffic Engineering, Jiangsu University, Zhenjiang 212013, China; 2222104147@stmail.ujs.edu.cn (G.Z.); 3200401204@stmail.ujs.edu.cn (Y.P.)

**Keywords:** deep learning, autonomous driving, instance segmentation, road traffic sign detection

## Abstract

With the rapid development of the autonomous driving industry, there is increasing research on related perception tasks. However, research on road surface traffic sign detection tasks is still limited. There are two main challenges to this task. First, when the target object’s pixel ratio is small, the detection accuracy often decreases. Second, the existing publicly available road surface traffic sign datasets have limited image data. To address these issues, this paper proposes a new instance segmentation network, RTS R-CNN, for road surface traffic sign detection tasks based on Mask R-CNN. The network can accurately perceive road surface traffic signs and provide important information for the autonomous driving decision-making system. Specifically, CSPDarkNet53_ECA is proposed in the feature extraction stage to enhance the performance of deep convolutional networks by increasing inter-channel interactions. Second, to improve the network’s detection accuracy for small target objects, GR-PAFPN is proposed in the feature fusion part, which uses a residual feature enhancement module (RFA) and atrous spatial pyramid pooling (ASPP) to optimize PAFPN and introduces a balanced feature pyramid module (BFP) to handle the imbalanced feature information at different resolutions. Finally, data augmentation is used to generate more data and prevent overfitting in specific scenarios. The proposed method has been tested on the open-source dataset Ceymo, achieving a Macro *F*_1_-score of 87.56%, which is 2.3% higher than the baseline method, while the inference speed reaches 23.5 FPS.

## 1. Introduction

In recent years, with the rapid development of parallel computing technology based on image data and the rapid iteration of sensors, research on autonomous driving perception algorithms has been greatly promoted. The information that the environmental perception system of the smart car needs to extract is the dynamic and static obstacles and the road surface environment. Dynamic obstacles mainly refer to other vehicles around the vehicle, pedestrians, and dynamic or static objects on the ground that may affect the safe driving of the vehicle; road pavement environment mainly refers to the geographical environment around the vehicle and road traffic information, such as lane lines, road traffic signs, etc. Among them is the accurate detection of traffic signs on the road surface, especially the accurate perception of straight arrows, left arrows, right arrows, straight-left arrows, straight-right arrows, pedestrian crossings, and slow signs, which is beneficial for intelligent vehicles to make normative braking during driving and, when combined with high-precision maps, can assist in path planning.

Research on traffic sign detection on road surfaces can be roughly divided into two categories: one is manual feature methods and object detection methods based on deep learning. Most of the traditional methods for road traffic sign detection manually extract the basic features of the target object, such as color, edge, and texture, which largely rely on the method designed by the authors. For example, Tang et al. [1] utilized histograms of oriented gradients (HOG) [2] and support vector machines (SVM) [3] with region of interest (ROI) constraints, which demonstrated good performance on the dataset. Compared with manual methods, deep learning-based methods show better results and stability in the feature extraction of road signs.

In deep learning-based computer vision, classification can be defined as predicting the class of an object in an image or providing a list of classes of objects in an image based on its classification score. Object detection or localization is a gradual process from coarse inference to fine inference, which not only provides the category of the image object but also gives the location of the classified image object in the form of a bounding box or center. The purpose of semantic segmentation is to obtain accurate inference results by predicting the label of each pixel in the image. Each pixel is classified and labeled according to the object or region in which it is located. Instance segmentation is essentially a more fine-grained visual perception task that combines two classic computer vision environment understanding subtasks: object detection and semantic segmentation. Therefore, instance segmentation can not only distinguish pixel categories but also accurately segment different instance objects belonging to the same semantic category, thereby providing rich perceptual information for downstream decision planning and other tasks. In the actual autonomous driving scene, for the detection task of road traffic sign the autonomous driving perception system not only needs to accurately identify the position of each type of road traffic signs, but also needs to accurately classify each pixel on the road traffic sign area. Therefore, using the instance segmentation method to detect road traffic signs can not only obtain the positions of different traffic signs but also classify each type of traffic sign at the pixel level.

However, the pixel ratio of road traffic signs in traffic scenes is relatively small, and their features are relatively sparse, which greatly limits the accuracy of instance segmentation algorithms. In addition, the public data for the road traffic sign detection task is very scarce, and most of the public data sets are small data sets, and the instance segmentation algorithm needs a lot of training to have better robustness. Based on these two challenges, this paper proposes the RTS R-CNN instance segmentation network, which is improved on the basis of Mask R-CNN [4]. The feature extraction network used by Mask R-CNN is ResNet [5]. Although the network stacks many convolutional layers, its effective depth is low, which easily leads to poor detection performance of small target objects, so we use efficient channel attention. The module ECA [6] further optimizes CSPDarknet53 and proposes CSPDarkNet53_ECA to replace the feature extraction module in the original network. At the same time, in order to improve the defects of FPN [7] and further improve the detection accuracy of the network for small target objects, we propose GR-PAFPN on the basis of PAFPN [8] and use the residual feature augmentation module (RFA) [9] to improve the top-down first-level features. Indicates that atrous spatial pyramid pooling (ASPP) [10] is introduced to obtain multi-scale feature information, and the balanced feature pyramid module (BFP) is used to enhance the expressive ability of each level of feature maps. Aiming at the small amount of road traffic sign data, we flipped and adjusted the color space on the training set pictures to increase the number of images from the small data set and changed the pixel coordinates of the flipped pictures.

In summary, the main contributions of this paper are as follows: Firstly, we proposed CSPDarkNet53_ECA to replace the original feature extraction module in the network. It enhances the channel features of the input feature map and improves the performance of deep convolutional networks by adding cross-channel interactions. Secondly, we proposed GR-PAFPN, which optimizes PAFPN using Residual Feature Aggregation (RFA) modules and Atrous Spatial Pyramid Pooling (ASPP) and introduces the Balanced Feature Pyramid (BFP) module to handle imbalanced information in different resolutions, thus improving the detection accuracy of small objects in the network. Finally, we increased the number of images from a small dataset by using data augmentation techniques such as flipping and color space adjustment, which helps to avoid overfitting specific scenarios.

## 2. Related Works

In this part, we review the road traffic sign detection algorithm based on deep learning and the instance segmentation algorithm, respectively.

### 2.1. Road Traffic Sign Detection

With the further development of deep learning, CNN-based object detection significantly improves the performance of road traffic sign detection. A convolutional neural network model is used in [11] that combines ResNet-101 [5] and pyramid pooling ensembles to obtain lane and road sign semantic segmentation output. Their architecture achieves average results on the TRoM [11] dataset, which can be considered a performance baseline. VPGNet [12] is an end-to-end model based on CNN architecture for simultaneous detection of lanes and road traffic signs. They regard pavement traffic sign detection as a grid regression task and then use grid sampling and box clustering as post-processing techniques to merge grid cells. However, they focus more on lane detection and vanishing point prediction tasks and only provide experimental results for four pavement traffic sign categories. It is worth mentioning that the authors released a new dataset that was publicly collected under various weather conditions in Korea. Hoang et al. [13] detected and classified arrow and bicycle markings on the road based on adaptive ROI and RetinaNet [14]. The results show that Adaptive ROI outperforms other methods. In pursuit of real-time detection, Zhang et al. [15] propose a method consisting of three modules: preprocessing, road traffic sign detection, and segmentation. In the second stage, a lightweight network combined with a Siamese attention mechanism is employed to improve accuracy and enhance sensitivity to road traffic signs. For the segmentation module, the segmented objects can achieve pixel-level accuracy at a lower computational cost. Ye et al. [16] proposed a two-stage model combining YOLOv2 [17] with a spatial transformation network (STN) [18] to address the distortion of road markings. Even for two-stage models, the proposed method achieves good performance with less computation. In conclusion, deep learning-based methods are more robust and stable than traditional feature extraction methods and can be applied to different scenarios with higher accuracy.

### 2.2. Instance Segmentation Algorithm

Instance segmentation algorithms based on deep learning can be mainly divided into four mainstream technical routes [19]: bottom-up methods based on semantic segmentation, detection-based methods, direct instance segmentation methods, and query-based methods. Semantic segmentation-based methods include SGN [20], which first generates segmentation masks with fixed semantic categories by pixel-by-pixel classification and then groups pixels into individual instances by means of clustering, metric learning, etc. This method can better retain the shallow, fine-grained features and geometric shape information, but its post-processing operation is more cumbersome, and the accuracy rate is low in complex working conditions with large objects and many categories. In order not to rely on methods such as bounding boxes or pixel embeddings, the direct instance segmentation method uses PolarMask [21] by directly predicting instance masks and semantic categories. Based on the intuition that the center position and size of the instance object are different, the SOLO series [22,23] divides the mask according to the image position. Specifically, the input image is divided into several grids, and the grid unit where the center of the instance is located is responsible for assigning a category to each pixel of the instance. Although this method effectively improves the detection speed, when the centers of multiple objects fall on the same grid, the predicted mask and category will be confused, which will affect the driving safety of autonomous vehicles. Detection-based methods first locate prior instances via powerful detectors [24,25,26] and then perform segmentation within the resulting regions of interest [27,28,29,30]. Thanks to the development of object detection, such methods are usually more accurate. The query-based approach utilizes the Transformer mechanism and expresses the object of interest through a query. ISTR [31] is the first end-to-end instance segmentation using low-dimensional mask embeddings.

According to the required order of object localization and mask generation, current detection-based instance segmentation methods can be divided into two types: single-stage methods and two-stage methods. The two-stage algorithm based on the region candidate network has high accuracy, but the small batch training of the model consumes a lot of memory resources and takes a long time for reasoning, such as Cascade Mask R-CNN [32]. A single-stage algorithm such as YoLACT++ [33], which performs localization and segmentation at the same time, has a faster inference speed, but its detection accuracy is obviously lacking.

## 3. Methodology

**Figure 1:**
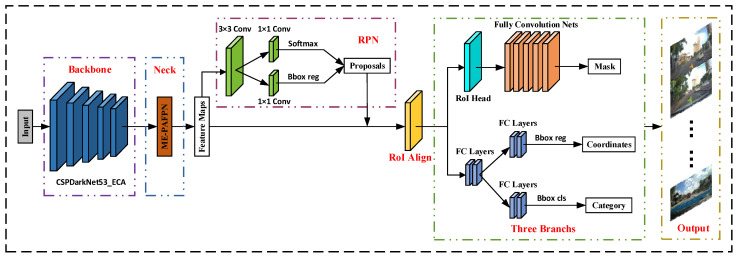
The network framework diagram of RTS R-CNN.

The instance segmentation network Mask R-CNN is mainly composed of the feature extraction module, the feature fusion module, the region candidate module RPN, and the prediction module. The road traffic sign detection algorithm model RST R-CNN proposed in this paper is based on Mask R-CNN. The feature extraction module and the feature fusion module are optimized separately. Specifically, the feature extraction network ResNet50 of the original model is replaced by the CSPDarkNet53, and on this basis, an Efficient Channel Attention module (ECA) is integrated. Considering that road traffic signs account for fewer pixels in the original image problem, we improve the Channel Enhanced Feature Pyramid Network PAFPN by enhancing the information flow and propose the GR-PAFPN module to replace the feature extraction network FPN in the original model. As shown in Figure 1, the network framework diagram of RTS R-CNN.

### 3.1. Backbone

**Figure 2:**
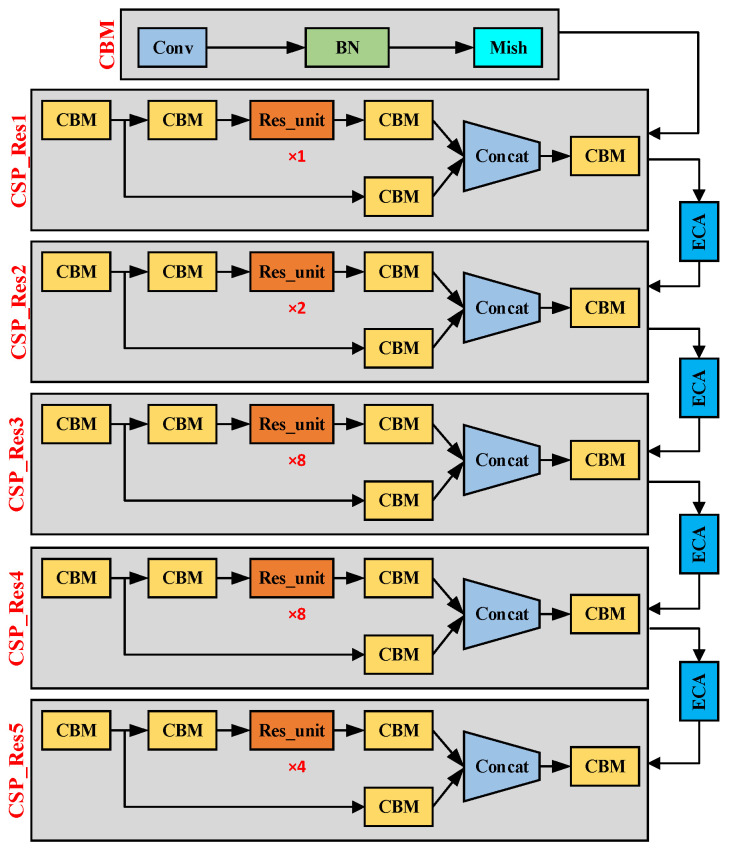
The structure of CSPDarknet53_ECA.

CSPDarknet53 has demonstrated excellent feature extraction capabilities in YOLOv4. It mainly includes 5 CSP_Res modules combined with a cross-stage partial network (CSP) and a residual network. It uses its large residual edge to expand the receptive field and Integrating local context information can improve the feature extraction ability of the convolutional network without losing detection accuracy, improve detection accuracy, and at the same time reduce the calculation loss of the entire model. Since road traffic signs are small targets, this puts high demands on the detection accuracy of the network model, so we use the efficient channel attention module ECA to further optimize CSPDarknet53. As shown in Figure 2, the structure diagram of CSPDarknet53_ECA. We add four high-efficiency channel attention modules ECA between the input and output of five CSP_Res modules and strengthen the channel features of the input feature map by increasing cross-channel interaction. Improving the performance of deep convolutional networks.

ECA removes the fully connected layer on the basis of the Squeeze-and-Excitation Network (SENet) and uses a 1 × 1 convolutional layer directly after the global average pooling layer so as to avoid learning channel attention information. The time channel dimension is reduced, and the number of parameters is reduced. In the conventional convolution operation, the size of the convolution kernel will affect the size of the receptive field. In order to extract different ranges of features from different input feature maps, ECA uses a dynamic convolution kernel to do 1 × 1 convolution to learn between different channels. importance. The term dynamic convolution kernel means that the size of the convolution kernel is adaptively changed through a function. In a layer with a large number of channels, a larger convolution kernel is used to perform 1 × 1 convolution, which enables more cross-channel interaction. In the layer with a small number of channels, a smaller convolution kernel is used to perform 1 × 1 convolution, so that there is less cross-channel interaction. The convolution kernel adaptive function is defined as follows:

(1) $$k=\Phi (C)={\left|\frac{\log_{2}(C)}{\gamma }+\frac{b}{\gamma }\right|}_{odd}$$

where, *k* indicates the size of the convolution kernel; *C* indicates the number of channels; ∥_odd indicates that *k* can only take odd numbers; γ, b=1.

**Figure 3:**
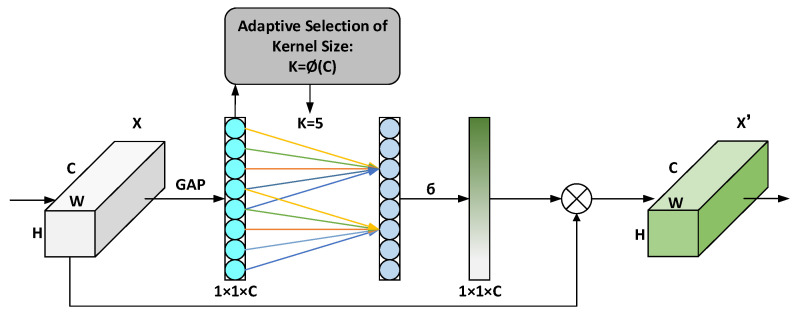
The structure of ECA module.

A schematic diagram of the structure of the ECA attention module is shown in Figure 3. The specific implementation process is: (1) first input the feature map, and its dimension is H × W × C; (2) perform spatial feature compression on the input feature map and use the global average pooling GAP in the spatial dimension to obtain 1 × 1 × C feature map; (3) Convolve the compressed feature map through 1 × 1 to learn the importance between different channels. At this time, the output dimension is still 1 × 1 × C; (4) Finally, the channel Attention combination, the feature map 1 × 1 × C of channel attention, and the original input feature map H × W × C are multiplied channel by channel, and finally the feature map with channel attention is output.

### 3.2. Feature Fusion Module

Mask R-CNN uses FPN as a feature extraction network. FPN has the following problems with the fusion of information features: (1) The information on the feature map is lost. FPN is a top-down propagation method. From the low-level feature map to the highest feature map, it must go through multiple network layers, and the network increases the difficulty of obtaining the initial image. (2) Multi-scale information is lost. FPN does not fully consider the difference in semantic information between different layers. Direct fusion between different features will reduce the ability for multi-scale feature representation. (3) The semantic information of non-adjacent layers cannot be fused. In FPN, only the semantic information of adjacent layers can be directly fused, and the semantic information of non-adjacent layers is diluted, which easily leads to the problem of imbalanced semantic features.

**Figure 4:**
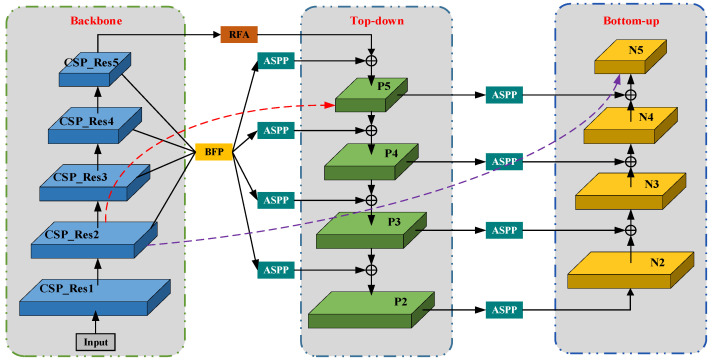
The structure of GR-PAFPA.

As shown in Figure 4, since a high response to object edges or instance parts is a strong indicator for accurately locating instances, PAFPN enhances the overall feature layer by constructing a bottom-up feature fusion network that reduces the propagation path of the main feature layer. Positioning ability: the red dotted line indicates that in the FPN network, the main feature map passes through multiple network layers from top to bottom, and the information of the main feature map is seriously lost. The purple dashed line represents the bottom-up feature fusion. Shallow features are connected to P2 by fusing the raw FPN at the bottom layer and then transferred from P2 to the top layer with bottom-up feature fusion. The number of layers is less than 10, which can better preserve shallow feature information. Although PAFPN improves the problem of loss of main feature information, it does not solve the problem of multi-scale information loss in FPN, and non-adjacent layer semantic information cannot be directly fused. To solve the problem of multi-scale information loss, we use Residual Feature Augmentation (RFA) and Atrous Spatial Pyramid Pooling (ASPP) to optimize PAFPN; in order to balance semantic features, we introduce the balanced feature pyramid Module (BFP) to deal with unbalanced information in each resolution.

#### 3.2.1. Residual Feature Augmentation Module

**Figure 5:**
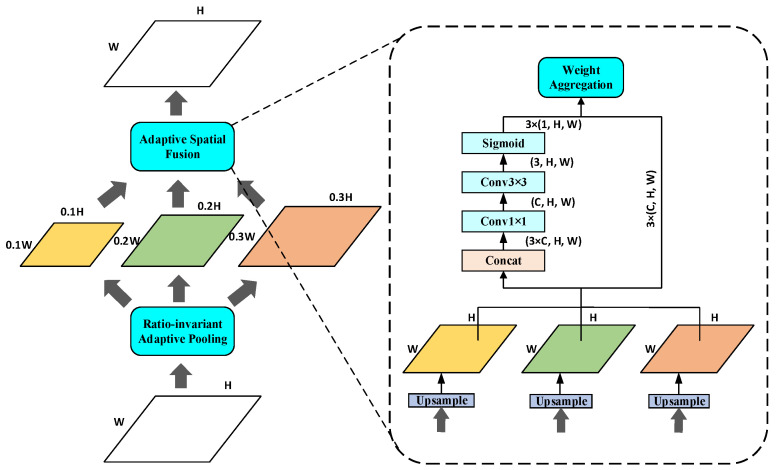
The structure of RFA.

In FPN, the dimensionality reduction operation is performed when the low-level features are fused from the highest layer to reduce the channel features, resulting in the loss of multi-scale information. To make up for this shortcoming, we use RFA to improve the top-down, one-level feature representation and fuse the feature information without information loss in CSP_Res5 into P5. As shown in Figure 5, firstly, the feature map output by CSP_Res5 is divided into three feature layers of different scales using Ratio-invariant Adaptive Pooling (RAP), and the scales are 0.1 times, 0.2 times, and 0.3 times that of the input feature map. Each feature map is then passed through a 1 × 1 convolutional layer, the number of channels C is reduced to 256, and finally bilinear interpolation is used for upsampling to restore the feature map to the scale size of the original input feature map. Considering the aliasing effect caused by interpolation, Adaptive Spatial Fusion (ASF) is used to adaptively combine the generated spatial weight probability map and the upsampled hierarchical feature map so that the output feature map has multi-scale feature information.

#### 3.2.2. Atrous Spatial Pyramid Pooling

**Figure 6:**
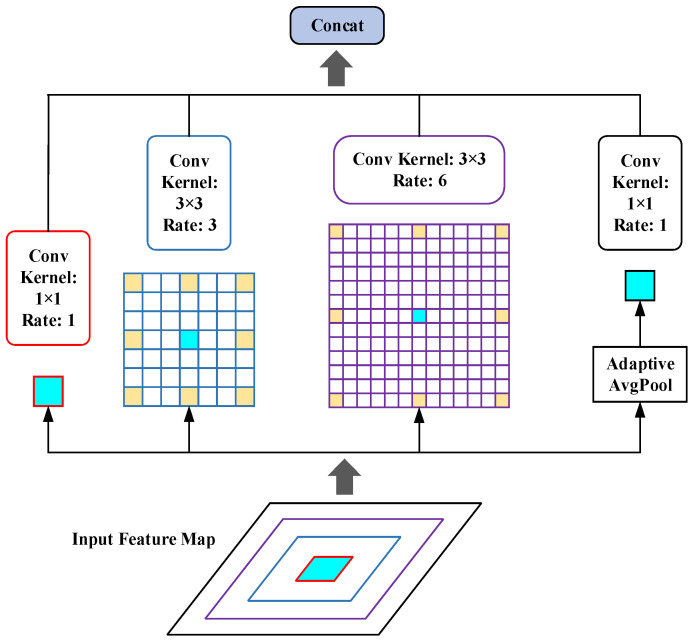
The structure of ASPP.

FPN reduces the number of channels of the feature map through a 1 × 1 convolution operation, which also suppresses the representation of multi-scale features, so we introduce ASPP to obtain multi-scale feature information. As shown in Figure 6, the four parallel branches of ASPP generate images of different scales through atrous convolution [34], using different atrous convolution rates and convolution kernels, where the convolution rates are 1, 3, and 6, respectively, and the convolution kernels sizes are 1 × 1 and 3 × 3, respectively. Using the ASPP module to improve the model’s performance in the fusion stage of each branch effectively expands the receptive field of feature images and improves the network’s ability to extract abstract information.

#### 3.2.3. Balance Feature Pyramid Module

**Figure 7:**
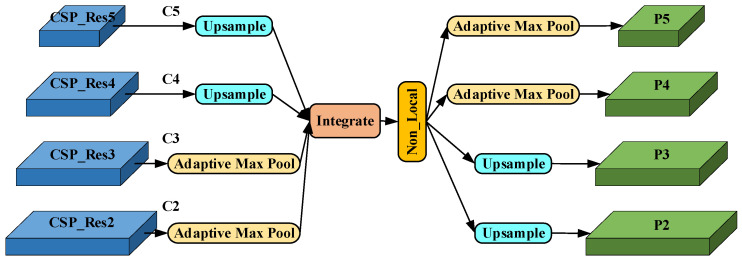
The structure of BFP.

High-level semantic information and shallow detail information can complement each other to improve the target detection effect, and the fused features need to deal with unbalanced information at each resolution. Therefore, we introduce the BFP module to scale, integrate, and refine the four-level feature maps and use the feature map information of multiple levels to enhance the expressive ability of each level of feature maps and realize the ability to deal with unbalanced information. As shown in Figure 7, in order to aggregate multi-level features, upsampling interpolation is used for small-size feature layers, adaptive maximum pooling is used for large-size feature maps, and multi-level features $\left\{C_{2},\;C_{3},\;C_{4},\;C_{5}\right\}$ are adjusted To $C_{4}$ size, a balanced semantic is obtained by simple integrating, which can be expressed as:

(2) $$\;\mathrm{Integrate}=\frac{1}{L}\sum_{l=l_{\min}}^{l_{\max}}C_{l}$$

where, *L* represents the total number of layers of the predicted feature layer, and the level of the predicted feature layer is defined as $C_{l}$.

**Figure 8:**
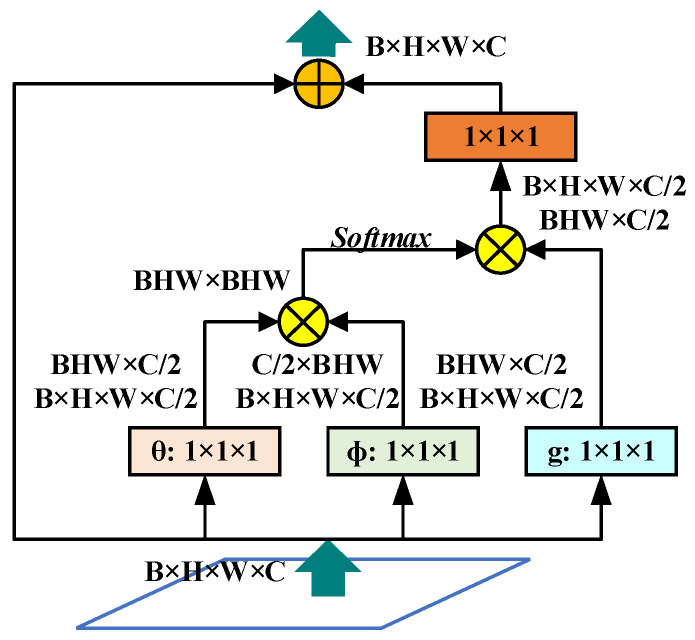
The structure of non-local module.

Then the non-local network [35] is used to further optimize the balance semantics to enhance the fusion features. As shown in Figure 8, first linearly map the input feature map to obtain the three features θ, ∅, g after channel compression; then perform feature merging through the reshape operation; then perform matrix point multiplication on θ and ∅ and use the activation function Get the weight, that is, the attention coefficient; then multiply it with the feature g to expand the number of channels; finally, sum the residual with the input feature map and output it.

After the non-Local module, we rescale the obtained features using the same but reversed process of semantic balancing to enhance the original features. During this process, each predictive feature layer obtains the same information from the other predictive feature layers.

### 3.3. Data Augmentation

Data augmentation is a common method for deep learning models that aims to increase the number of images from small datasets to avoid overfitting specific scenarios. This section will detail data augmentation on images. Data augmentation on labels due to changes in pixel coordinates will also be addressed in this section.

#### 3.3.1. Data Augmentation on Image

Deep learning networks usually require a large amount of training data to achieve better results. In the case of limited data acquisition, data augmentation techniques are used to generate more data from existing datasets, thereby increasing the diversity of original images and making up for the lack of data. Common techniques for data augmentation include: (1) geometric transformation: randomly flip, crop, rotate, shear, or translate the image, (2) color space transformation: change the color channel space or try to map RGB to other color spaces, (3) Noise injection: A matrix of random values sampled from a Gaussian distribution is added to the pixels of the image; (4) Kernel filter: A kernel filter on the image for convolution operations such as sharpening and blurring. After data augmentation, what people see with their eyes is still easily recognizable as the same image, but to the deep learning model, these processed images are completely new images.

Considering the simple features and monotonous colors of road traffic signs, they do not contain diverse structural features for object detection models. Therefore, this paper uses flipped and color space-adjusted (brightness and contrast) training datasets. Flipping is an effective approach and has been shown to be useful for improving the performance of deep learning models. Furthermore, color space adjustments are the easiest and most common technique for changing the brightness of an image. In the road surface environment, the diversity of lighting conditions and weather conditions has an impact on the accuracy of the model, so data augmentation is an important technique to change the image through color space adjustment. Flipping the image horizontally and vertically is a common method in geometric transformation. Additionally, brightness adjustment is implemented on the training data to transform brightness-related channels according to value settings. Therefore, it can make the image slightly brighter or darker to enhance the lighting conditions of the image. Contrast adjustment is also one of the data augmentation techniques used to rescale the range of intensity values in an image. Contrast is the ratio between the lightest and darkest areas of an image. The larger the ratio, the more shades of gray there are from black to white, which makes objects or boundaries in the image more distinguishable. As a result, the contrast of white road markings on black asphalt roads is enhanced, improving visual perception. Finally, quadruple image copies are generated using data augmentation techniques that increase the amount of data from the original image without adding additional time cost.

#### 3.3.2. Data Augmentation on Label

The ground-truth labels of target objects are crucial for supervised learning networks. Before training the model, it is necessary to label the target object labels of the dataset as ground truth. However, changing more original images through data augmentation requires a new labeling of the labels of the original images, which is a very time-consuming task. Therefore, this study increases the amount of data by performing data augmentation and homography transformation on images in a limited dataset. After data augmentation and homography transformation, annotations do not need to be manually labeled again. In view of the change in pixel coordinates, some labels of the augmented data need to be modified. After data augmentation such as brightness and contrast adjustments, the labels are the same as the original annotation files, while the pixel coordinates of flipped images need to be flipped horizontally. The pixel coordinates of the flipped image are transformed from the left part to the right part.

### 3.4. Loss Function during Training

The loss function used in this article mainly continues the loss function in Mask R-CNN, namely:

(3) $$L=L_{cls}+L_{box}+L_{mask}$$

They are the classification loss $L_{cls}$, the regression box loss $L_{box}$ and the mask loss $L_{mask}$. The first two losses are consistent with the object detection network Fast-RCNN [36]. The calculation formula of $L_{mask}$ is as follows:

(4) $$L_{mask}=\frac{1}{m^{2}}\sum_{i}^{k}\left(1^{k}\right)\sum_{1}^{m^{2}}[-y\log sigmoid(x)-(1-y)\log(1-sigmoid(x))]$$

where, $1^{k}$ means that when the kth channel corresponds to the true category of the target, it is 1, otherwise it is 0; *y* means the label value of the mask at the current position; the output value of the current position of *x*, *sigmoid(x)* means that the output x has passed through sigmoid The result after the function transformation; $m^{2}$ represents the dimension.

## 4. Experiments and Results

### 4.1. Introduction to Public Dataset

The public dataset used in this experiment is Ceymo [37], a dataset for road traffic sign detection, which consists of 2887 images, of which 4706 road sign instances belong to 11 categories. The images have a high resolution of 1920 × 1080 and capture a wide range of traffic, lighting, and weather conditions. This dataset uses the labelme annotation tool [38] to manually annotate road markings belonging to 11 categories into polygons. Each image has a JSON file that contains the coordinates of the polygons that enclose the road markings in that image. In addition to polygon annotations in JSON format, bounding box annotations in XML format and pixel-level segmentation masks in PNG format are provided to facilitate different road marking detection methods.

This dataset uses *F*_1_-score and Macro *F*_1_-score as rating indicators. Calculate the IoU between the prediction and the road truth. When the IoU is greater than 0.3, the corresponding prediction is regarded as the real prediction. The total number of true positives (TP), false positives (FP), and false negatives (FN) is used to calculate precision, recall and *F*_1_-score as follows:

(5) $$\;\mathrm{precision}=\frac{TP}{TP+FP}$$

(6) $$\;\mathrm{recall}=\frac{TP}{TP+FN}$$

The Macro *F*_1_-score is calculated as the average of the individual *F*_1_-score of the 7 categories in the dataset, as follows:

(7) $$\;\mathrm{Macro}\;F_{1}-\mathrm{score}=\frac{1}{C}\sum_{i=1}^{C}F_{1}{-\mathrm{score}}_{i}$$

where, *C* represents different classes in the dataset, and the Macro *F*_1_-score has the same importance for all classes, no matter how frequently they appear in the dataset.

### 4.2. Experimental Details

Instance segmentation models perform a lot of complex matrix operations and floating-point operations during training to find the optimal solution. The instance segmentation model proposed in this paper for road traffic sign detection uses the Pytorch1.13.1 deep learning framework for distributed data parallel training and synchronous batch normalization based on 64-bit NVIDIA (NVIDIA Co., Ltd., Santa Clara, CA, USA) and Ubuntu20.04 (Canonical Co., Ltd., London, UK), with two GTX3090 graphics cards (NVIDIA Co., Ltd.). We use stochastic gradient descent (SGD) as the optimizer, with the weight decay rate set to $4\times 10^{-4}$ and the momentum set to 0.9.

The choice of learning rate, batch size, and loss function affects the training speed of the segmentation model and the final segmentation accuracy. A higher learning rate will lead to a sharp increase in the loss while increasing the training speed, so it is more suitable to use a higher learning rate in the initial stage of training. If the learning rate is set too low, the convergence speed of the model will be slower, and it will be easier to find the optimal solution of the model, but the model will appear overfitting, so setting a smaller learning rate in the later stages of training will be more suitable. Considering the appeal factor, we use a multi-adaptive learning rate, that is, the learning rate will continue to decrease as the number of iterations increases, which is expressed as follows:

(8) $$l_{r}=\ln l_{r}{\left(1-\frac{\;epoch\;}{\;epoch_{\max}}\right)}^{power}$$

where, $lnl_{r}$ represents the initial learning rate, which is set to 0.1; epoch represents the number of current epochs; ${epoch}_{max}$ represents the maximum number of epochs, which is set to 150; and power represents exponential decay, which is set to 1. Similarly, the size of the batch setting will also affect the training speed and segmentation accuracy; if the batch size is too large, the training speed will be significantly improved, but the utilization of GPU memory will be affected, resulting in a decrease in segmentation accuracy. In this paper, we will set the batch size to 6.

### 4.3. Experimental Results

In this section, we compare the results of the proposed model on the dataset Ceymo with some of the current advanced methods, including MaskLab [39], Cascade Mask R-CNN, RetinaNet, and YoLACT++. This paper chooses Mask R-CNN as the baseline model (Baseline). The first two grids are instance segmentation methods based on a two-stage framework, and the quality of their position mask generation is highly dependent on the object localization network. The latter two networks are instance segmentation methods based on a single-stage framework. Using the global mask does not require the processes of clipping and RoI Align, but directly predicts an instance from the entire feature map. The model proposed in this paper is based on a two-stage framework. For the reliability of the experimental comparison, we use networks based on different frameworks for comparison. The inference speeds of all the above algorithms follow the default configuration, and the comparison results are shown in Table 1.

It is observed that the instance segmentation model based on the two-stage framework has an overall better instance segmentation effect than the single-stage method, but the network reasoning speed is significantly slower. Compared with the compared network, the instance segmentation performance of the method proposed in this paper is excellent overall, and the Macro *F*_1_-score can reach 87.14%, especially in targets with relatively small proportions of pixels, such as going straight, turning left, and decelerating signs. *F*_1_-score reached 89.31%, 75.36%, and 94.34%, respectively. It is obvious that our network model has more advantages, for instance, for the segmentation effect of small targets. In terms of inference speed, although it is slightly lower than YoLACT++ based on the single-stage framework, it has a greater advantage compared with the two-stage instance segmentation network.

**Figure 9:**
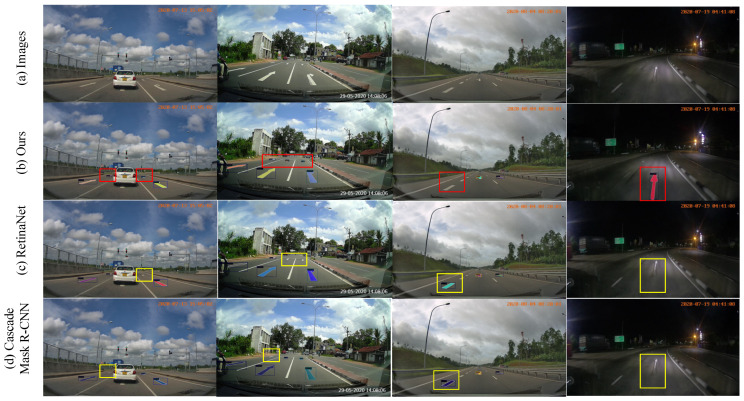
Comparison chart of visualization results on the Ceymo dataset.

**Figure 10:**
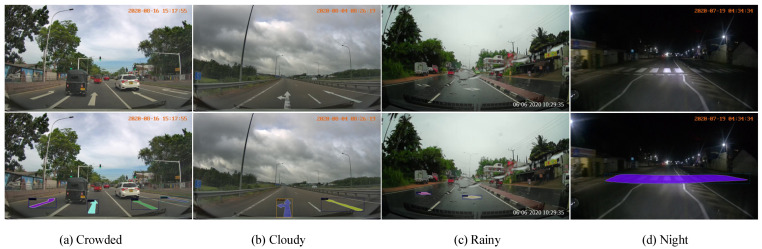
Visualization in extreme scenarios.

In order to further verify the effectiveness of the proposed method, we selected 4 pictures from the verification set for visualization, and the results are shown in Figure 9. It can be seen that the method proposed in this paper works well in congested, cloudy, rainy, and dark scenes. A better instance segmentation effect is obtained. At the same time, we also visually compared our method with the RetinaNet of the single-stage framework and the Cascade Mask R-CNN of the two-stage framework. The results are shown in Figure 10. It can be seen intuitively that there are small The problem of missed detection of targets and false detection of large targets, as well as the target of worn-out ground traffic signs cannot be accurately identified. Qualitative results show that the proposed method significantly improves the misdetection of large-sized traffic signs, reduces the misdetection rate of small traffic signs in the foreground area to a certain extent, and cannot accurately predict the wear and tear of traffic signs.

### 4.4. Ablation Experiment

In order to verify the effectiveness of each component module of the proposed method, we use Mask R-CNN as the Baseline to add each module in turn and test them on the Ceymo verification set, respectively. The performance results are shown in Table 2. By replacing the feature extraction network ResNet in the original network with CSPDarknet53_ECA, the Macro *F*_1_-score of the model increased by 0.81%, which effectively verified that integrating the ECA attention module into CSPDarknet53 as the backbone network can effectively improve the detection and segmentation performance of the model. Replace the feature fusion module FPN in the original network with GR-PAFPN. This feature fusion is designed and integrated into the original PAFFN to incorporate the residual feature augmentation module RFA, the atrous spatial pyramid pooling ASPP, and the BFP that can balance semantic features. The Macro *F*_1_-score of the model It has been improved by 0.16%, 0.79%, and 0.09% in turn, which proves that the feature fusion module we designed improves the problem that the information of traditional FPN feature maps and multi-scale information is easily lost and avoids the non-fusion of semantic information in non-adjacent layers. Finally, data enhancement was used, and the Macro *F*_1_-score of the model was increased by 0.55%, which proved that flipping and color space adjustment on images and labels can effectively improve the detection and segmentation effects of the model.

## 5. Conclusions

This paper proposes an instance segmentation framework, RTS R-CNN, based on the Mask R-CNN algorithm for road traffic sign detection tasks. The CSPDarknet53_ECA feature extraction network is proposed, which further optimizes CSPDarknet53 using the efficient channel attention module to strengthen the channel features of the input feature maps and improve the performance of deep convolutional networks by increasing cross-channel interaction. To improve the detection accuracy of small targets, GR-PAFPN is proposed in the feature fusion part, which optimizes PAFPN using the Residual Feature Aggregation module (RFA) and the Atrous Spatial Pyramid Pooling (ASPP) and introduces a Balanced Feature Pyramid module (BFP) to handle the imbalanced feature information in various resolutions. To address the issue of small road traffic sign image datasets, data augmentation techniques such as flipping and color space adjustment are used to increase the number of images from small datasets and prevent overfitting of algorithms to specific scenarios. The results on the publicly available Ceymo dataset demonstrate that the proposed algorithm is significantly better than the original method, with a Macro *F*_1_-score of 87.56%, which is 2.3% higher than the baseline method. Moreover, compared with other advanced instance segmentation networks, our proposed method significantly improves the false detection of large traffic signs and reduces the under-detection rate of small traffic signs in the far-field area to some extent. However, accurate predictions cannot be made for traffic signs that are heavily worn out. Future work will focus on improving the detection accuracy of these signs. Moreover, the issue of insufficient publicly available datasets for road traffic sign detection can be addressed by collecting a large number of images using onboard cameras on real transport vehicles and annotating them using professional annotation software. Since road traffic signs are small objects with a low pixel ratio, attention mechanisms can be designed in the future to improve the segmentation performance of small targets. This would help to further enhance the detection accuracy of small road traffic signs.

## Figures and Tables

**Table 1:** Comparison results with mainstream schemes on the Ceymo dataset.

Category	Masklab	Cascade Mask R-CNN	RetinaNet	YoLACT++	Ours
Straight Arrow	87.51	87.39	86.98	87.23	89.31
Left Arrow	74.27	73.97	72.11	71.06	75.36
Right Arrow	90.64	91.93	90.21	89.47	91.32
Straight-Left Arrow	80.21	81.92	79.39	80.11	82.69
Straight-Right Arrow	82.51	79.06	78.72	81.49	80.11
Slow	95.56	95.70	96.01	93.38	94.34
Pedestrian Crossing	94.95	95.44	93.56	92.61	96.86
FPS	14.2	13.3	23.6	24.0	23.5
Params(M)	75.32	77.1	37.74	35.29	36.85
Macro *F*_1_-score	86.52	86.49	85.28	85.05	87.56

**Table 2:** Ablation experiments on the Ceymo dataset. “√” indicates that the current module was used in the ablation experiment.

Baseline	CSPDarknet53_ECA	RFA	ASPP	BFP	Macro *F*_1_-Score
√					85.26
√	√				85.97
√	√	√			86.13
√	√	√	√		86.92
√	√	√	√	√	87.56

## Data Availability

Not applicable.
